# *Cirsium semzinanicum* (Asteraceae), a new species from Hakkâri, Turkey

**DOI:** 10.3897/phytokeys.68.8745

**Published:** 2016-08-05

**Authors:** Mehmet Fırat

**Affiliations:** 1 Yüzüncü Yıl University, Faculty of Education, Department of Biology, TR-65080, Van, Turkey

**Keywords:** Asteraceae, Epitrachys, Cirsium, Hakkari, Turkey, taxonomy

## Abstract

*Cirsium semzinanicum* **sp. nov.** (Asteraceae) is described as a new species from Hakkâri, Turkey. The new species is a part of the sect. *Epitrachys* (Cardueae) and similar to *Cirsium karduchorum*, from which it differs in morphological characters such as leaves, involucre, phyllaries, corolla, achens and pollen morphology. Geographical distribution, habitat and IUCN conservation status of this species are given.

## Introduction

The genus *Cirsium* Mill. is one of the largest genera of Asteraceae, and it comprises more than 250 perennial, biennial, or rarely annual spiny species distributed in the northern hemisphere in Europe; North Africa; East, Central, and Southwest Asia; and North and Central America (Charadze 1963, Davis and Parris 1975, Petrak 1979, Kadereit and Jeffrey 2007).

The last revision on *Cirsium* species growing in Turkey was carried out by Davis and Parris (1975) for the Flora of Turkey. In that study, 52 species (65 taxa) were given under 3 sections [*Cirsium*, *Epitrachys* DC., and *Cephalonoplos* (Neck.) DC.]. Additional taxonomic treatments have dealt with the distribution of the genus in supplements and 5 new species (6 taxa) were given (Davis et al. 1988; Güner et al. 2000).

Finally, the Turkish members of the genus were established as 66 species (78 taxa) according to Yıldız (2012) and Yıldız et al. (2013).

Between 2011 and 2015 new populations of *Cirsium* sp. similar to *Cirsium karduchorum* were discovered at 3 sites in south-east Turkey. Here the morphological and micro-morphological characters of these new populations are presented ad their taxonomic treatment is proposed.

## Materials and methods

**Figure 1.**
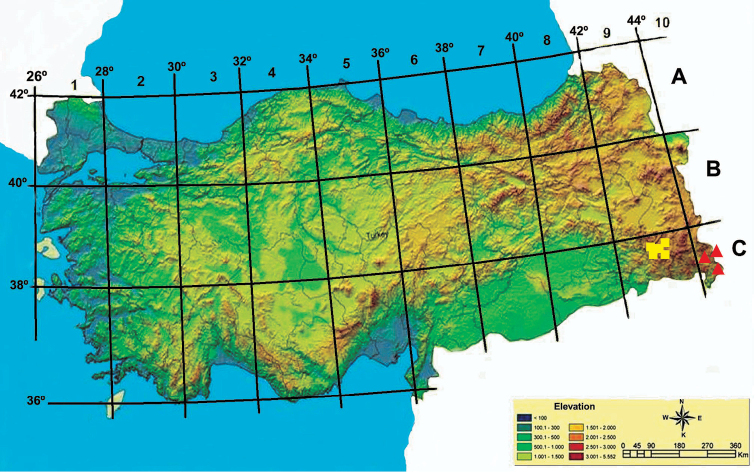
Distribution map of *Cirsium semzinanicum* sp. nov. (▴) and alsoclosely related species *Cirsium karduchorum* (●) in Turkey.

The specimens were collected by the author in July 2011 and August 2015 during the field trips to Şemdinli (Fig. 1) in Hakkâri, in the southeast of Turkey. In total 10 herbarium specimens of the presumed new species were collected from three adjacent localities and deposited in the herbaria of VANF, ANK and E. A formal name is provided accompanied by a detailed description and illustration.

At first glance, on the basis of the characters of leaves, involucre, phyllaries, corolla and achenes, seemed tobe similar to *Cirsium karduchorum*. The newly collected specimens were therefore cross-checked with the keys provided by de Candolle (1838), Boissier (1875), Petrak (1910), Davis and Parris (1975), Davis et al. (1988) and Güner et al. (2000). They were compared with the related specimens stored in VANF, GAZI, ANK and HUB herbaria.

Images of the collected material were taken with a Sony DSCR1, digital camera. The SEM micrographs were taken with a ZEISS supra55. The terminologies for pollen morphology were used in accordance with Faegri and Iversen (1989) and Punt et al. (1994). Geographical positions were identified using a Magellan explorist 710 GPS. According tothe grid system (Davis 1965), especially the new species which are present in Hakkâri province falls within the C10 square (Davis 1965). The conservation status of the new species was assesed according toIUCN criteria (IUCN 2014).

## Taxonomy treatment

### Cirsium semzinanicum

Taxon classificationPlantaeAsteralesAsteraceae

Fırat sp. nov.

urn:lsid:ipni.org:names:77156986-1

Figs 2 , 3

#### Type.

Turkey. C10 Hakkâri: Şemdinli, Bêgoz village, Kaduna region, rocky slopes, eroded slopes, 37°17'15"N, 44°25'25"E, 1717 m, 26 July 2011, *M.Fırat 27257* (holotype VANF!, isotypes ANKA, E, and Hb. M. Fırat).

#### Diagnosis.

*Cirsium semzinanicum* clearly differs from *Cirsium karduchorum* Petr. in its stems 100‒150 cm tall (vs. 50‒100 cm tall, robust), basal leaves 15‒25 × 9‒14 cm “excluding 10‒18 petiole”, green, (vs. 30‒40 × 15‒20 cm “excluding 15‒20 cm petiole”, bluish-green), involucre 15‒25 × 20‒30 mm ovoid tosubglobose (vs. 25‒30 × (30‒)40‒50 mm depressed subglobose tobroadly obovoid), phyllaries 9‒12-seriate ± erecto-refIexed (vs. 11‒14-seriate refIexed or recurved), achen brownish 7‒9 × 3‒4 mm (vs. bright grayish brown 6‒7.5 × 3 mm).

#### Description.

Perennial, few stemmed from base, erect, 100‒150 cm tall, unwinged but longitudinally striate, glabrous‒glaucous, with few branches above, sterile shoots at the base. Basal leaves long‒petiolate, petioles 10‒18 cm long, winged, with large auricules; lamina broadly elliptic in outline, 15‒25 × 9‒14 cm, green, very sparsely spinulose‒strigose above with 0.5‒0.9 mm long, adpressed setae, ca 1‒3 per 2 mm square, otherwise densely arachnoid; lower surface very sparsely arachnoid, 3/4 pinnatifid with 4‒5‒pairred, 5‒8 × 2‒5 lateral and terminal lobes with stout 7‒12 mm apical spine. Middle cauline leaves similar but smaller tobasal leaves, petioles 1‒6 cm long. lamina broadly elliptic in outline 4‒8 × 2,5‒4 cm long, lateral and terminal lobes 3/4 pinnatifid with 3‒5 lobes, spinulose-strigose above with 0.7‒1.2 mm long, adpressed setae, ca 4‒6 per 2 mm square, otherwise densely arachnoid with stout 6‒9 mm apical spine. Upper cauline leaves similar middle cauline leaves but smaller and sessile. shortly auriculate. Uppermost (involucral) leaves 2‒3, 1‒3 cm long, linear‒lanceolate with sinuate‒dentate margin, shorter than involucre, sessile. Capitula erect, 1(‒2) on each branch, compound corymbose with 15‒30‒capitula, 26‒35 mm × 2‒30 mm, sessile or peduncles 1‒2 cm long; involucre 15‒25 × 20‒30 mm, ovoid tosubglobose; phyllaries 9‒12‒seriate, adpressed, glabrous, greensh‒yellowish, ± erecto‒reflexed, the outer 8‒10 × 2‒3 mm, with reflexed, ovate-oblong, 3‒5 × 0.3‒0.4 mm long apical spine; the median 10‒14 × 2‒3 mm long, with ±reflexed, oblong‒lanceolate, 4‒6 × 0.2‒0.3 mm long apical spine; the inner 16‒21 × 1‒2 mm, linear-lanceolate, 5‒7 × 0.1‒0.2 mm long apical spine. Corolla pinkish-whitish, 26‒32 mm long. lobed to1/4‒1/5. Style 15‒19 mm long, exerted, shortly bilobed; filaments 5‒7 mm long, densely hairy; Achenes brownish, 7‒9 × 3‒4 mm, oblong, asymmetric, slightly compressed. Pappus 16‒18 mm long, plumose, dirty white, or light brown.

**Figure 2.**
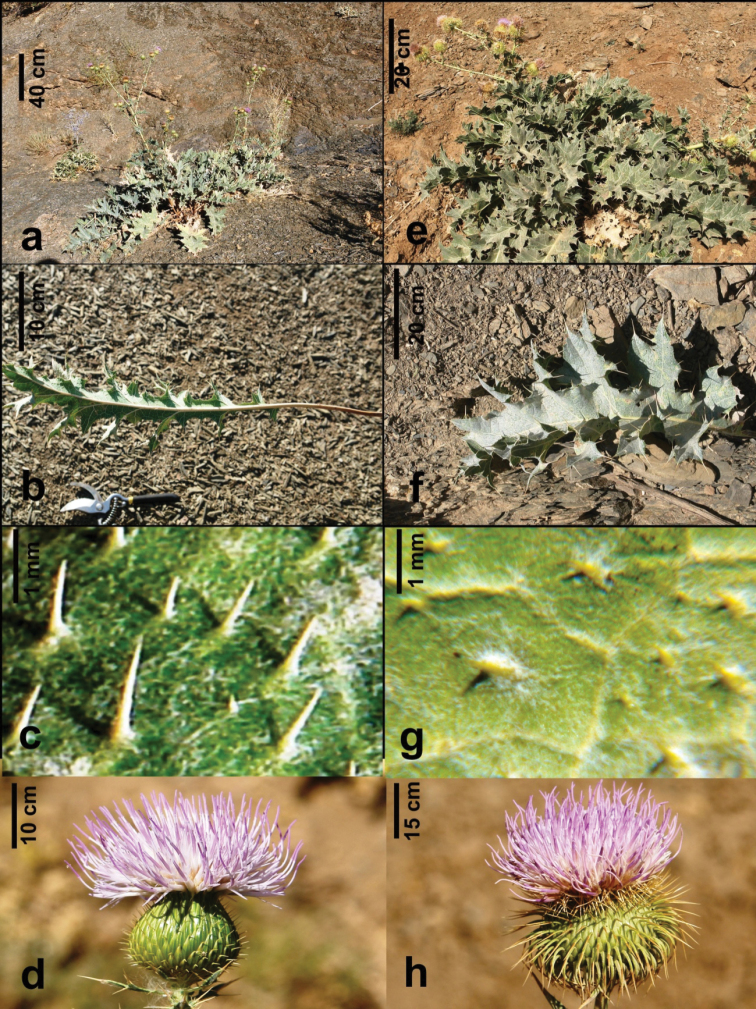
*Cirsium semzinanicum* sp. nov.: **a** habits **b** basal leaf **c** upper leaf surface **d** capitulum. *Cirsium karduchorum*: **e** habit **f** basal leaf **g** upper leaf surface **h** capitulum.

**Figure 3.**
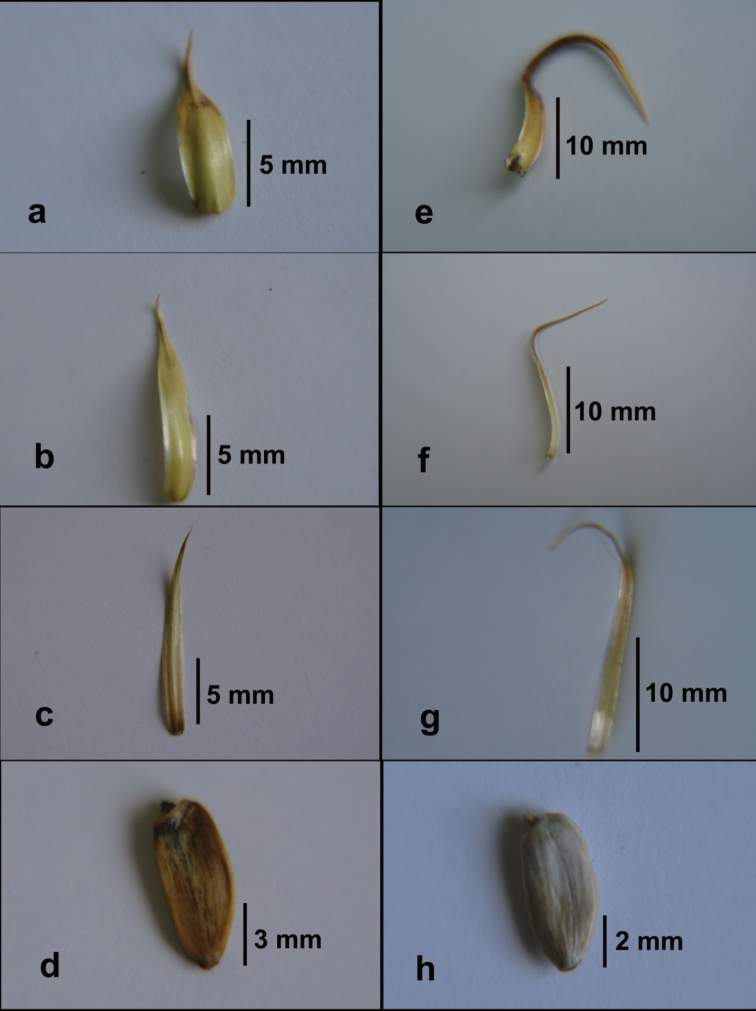
*Cirsium semzinanicum* sp. nov.: **a** outer phyllary **b** median phyllary **c** inner phyllary **d** achene. *Cirsium karduchorum*: **e** outer phyllary **f** median phyllary **g** inner phyllary **h** achene.

#### Micromorphology.

**Figure 4.**
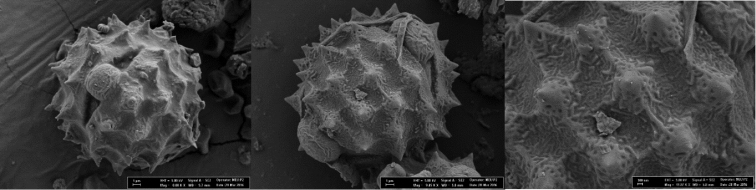
SEM Microphotograph of pollen of *Cirsium karduchorum*.

Pollen grains are tricolporate, pollen shapes P/E: spheroidal, amb circular, polar axis 36.72 µm, equatorial axis 36.60 µm, exine 1.62 µm thick, ornamentation echinate. Tectum complete structured, spines conic and pointed, 5-6 per 100 µm^2^, 2.83 µm long, base diameter 3.45 µm, intine 1.02 µm, colpi margins are straight and distinct with pointed ends, 10 µm long (Fig. 4).

**Figure 5.**
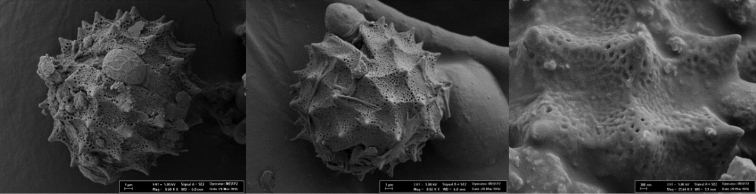
SEM Microphotograph of pollen of *Cirsium semzinanicum* sp. nov.

Pollen grains are tricolporate, pollen shapes P/E: spheroidal, amb circular, polar axis 36.21µm, equatorial axis 38.25 µm, exine 1.73 µm thick, ornamentation echinate, tectum complete structured, perforate with spines conic and pointed, 5–6 per 100 µm^2^, 2.70 µm long, base diameter 3.45 µm, intine 0.95 µm, colpi margins are straight and distinct with pointed ends, 17.5 µm long (Fig. 5).

#### Phenology.

Flowering and fruiting, from July to September.

#### Vernacular name.

*Cirsium* spp. are called as “Kîvar”by the local people of the Hakkâri province (Fırat 2013).

#### Etymology.

The specific epithet is derived from the name of the Şemzinan (Şemdinli) province where type material was collected.

#### Distribution and conservation status.

*Cirsium semzinanicum* is endemic to Hakkâri province in Turkey. The number of mature individuals is approximately 600 and is known from 3 locations (criteria B2ab [i.iii]). Therefore, it should be regarded as belonging tothe IUCN Vulnerable (VU) threat category (IUCN 2014).

#### Habitat and ecology.

The new species grows in Oak openings, slopes and eroded slopes at c. 1600–1900 m elevation with plants such as *Quercus libani*, Daphne oleoides subsp. kurdica, *Satureja bachtiarica*, *Dianthus orientalis*, *Echinops tournefortii*, *Astragalus* sp., *Eryngium billardierei*.

## Discussion

*Cirsium semzinanicum* is morphologically similar to *Cirsium karduchorum* because of having similar habitus, lesser involucre and phyllaries, corolla and achen size, but it is easily distinguished from its stems 100‒150 cm tall (not 50‒100 cm tall, robust), basal leaves 15‒25 × 9‒14 cm “exculiding 10‒18 petiole”, green, (not 30‒40 × 15‒20 cm “exculiding 15‒20 petiole”, bluish‒green), involucre 15‒25 × 20‒30 mm ovoid tosubglobose (not 25‒30 × (30‒)40‒50 mm depressed subglobose tobroadly obovoid), phyllaries 9‒12‒seriate ±erecto-refIexed (not 11‒14‒seriate refIexed or recurved), achen brownish 7‒9 × 3‒4 mm (not bright grayish brown 6‒7.5 × 3 mm). Additional morphological differences between the new species and *Cirsium karduchorum* are given in Table 1. Pollen morphology of both of species shows similarity. Pollen grains are isopolar, tricolporate, pollen shapes are spheroidal. Colpus length of *Cirsium semzinanicum* is longer than *Cirsium karduchorum*, while tectum completely structured, spines conic and pointed in *Cirsium karduchorum*, the tectum complete structured, perforate with spines conic and pointed in *Cirsium semzinanicum* (Figs 4 and 5).

## Additional specimens examined

*Cirsium semzinanicum*: Turkey. C10 Hakkâri: Şemdinli, above Benavok village, Gerdi şapatan region, rocky slopes, eroded slopes, 37°09'53"N, 44°26'00"E, 1692 m, 8 August 2013, *M.Fırat 30332* (VANF! and Hb. M. Fırat); C10 Hakkâri: Şemdinli, Berxoşe region, rocky slopes, 37°20'62"N, 44°34'64"E, 2072 m, 4 August 2014, *M.Fırat 30958* (Hb. M. Fırat)

*Cirsium karduchorum*: Turkey. C9 Hakkâri: Karadağ mountain above Hakkâri, eroded slopes, 2438 m, 13 August 1954, *Davis & Poluinin 24326* (ANK!, E! and W!); Hakkâri: from Hakkâri to Berçelan plateau, eroded slopes, 2150 m, 5 September 2007, *M. Fırat & T. Dirmenci 3579* (Hb. M. Fırat); Hakkâri: from Hakkâri to Berçelan plateau, eroded slopes, 37°36'00"N, 43°44'50"E, 2130 m, 16 August 2008, *M. Fırat, T. Dirmenci, B. Yıldız 16932* (Hb. M. Fırat); Hakkâri: Kotranis region, eroded slopes, 37°44'12"N, 43°44'51"E, 2277 m, 16 August 2011, *M. Fırat 27767* (Hb. M. Fırat); Hakkâri: from Hakkâri to Berçelan plateau, eroded slopes, 37°36'00"N, 43°44'50"E, 2130 m, 16 August 2013, *M. Fırat 30439* (Hb. M. Fırat).

## Supplementary Material

XML Treatment for Cirsium semzinanicum
